# Crystal structure and Hirshfeld surface analysis of (*E*)-2-[1-hy­droxy-2-(pyridin-2-yl)eth­yl]-4-[2-(4-meth­oxy­phen­yl)diazen-1-yl]phenol

**DOI:** 10.1107/S2056989019004377

**Published:** 2019-04-09

**Authors:** Md. Serajul Haque Faizi, Pratik Sen, Gyanesh Kumar Saxena, Irina A. Golenya

**Affiliations:** a Department of Chemistry, Langat Singh College, B. R. A. Bihar University, Muzaffarpur, Bihar-842001, India; b Department of Chemistry, Indian Institute of Technology Kanpur, Kanpur, UP-208016, India; c National Taras Shevchenko University, Department of Chemistry, Volodymyrska str., 64, 01601, Kyiv, Ukraine

**Keywords:** crystal structure, azo compounds, diazen­yl, pyridine, hydrogen bonding, C—H⋯π inter­actions, offset π–π inter­actions, supra­molecular framework, Hirshfeld surface analysis

## Abstract

In the title compound, C_20_H_19_N_3_O_3_, the configuration about the azo N=N bond is *E*, and the central benzene ring is inclined to the pyridine ring by 31.43 (8)° and to the 4-meth­oxy­phenyl ring by 4.73 (8)°. In the crystal, mol­ecules are linked by pairs of O—H⋯N hydrogen bonds, forming inversion dimers with an *R* ^2^_2_(12) ring motif. The dimers are linked by O—H⋯O and C—H⋯O hydrogen bonds, forming layers parallel to the *ac* plane. There are C—H⋯π inter­actions present within the layers and between the layers, leading to the formation of a supra­molecular framework. The layers are also linked by offset π–π inter­actions, with an inter­planar distance of 3.416 (2) Å.

## Abstract 2

In the title compound, the configuration about the N=N bond is *E*, and the central benzene ring is inclined to the pyridine ring by 31. 43 (8)° and to the 4-meth­oxy­phenyl ring by 4.73 (8)°. In the crystal, mol­ecules are linked by pairs of O—H⋯N hydrogen bonds, forming inversion dimers with an 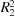
(12) ring motif.

## Chemical context   

Azo compounds have received much attention in fundamental and applied chemistry (Nishihara, 2004 ▸; İspir, 2009 ▸). The well-known applications of azo dyes in acid–base indicators and chemical sensors and as electron-transfer catalysts have attracted the inter­est of many investigators (Tunçel & Serin, 2006 ▸). The versatile applications of azo compounds in various fields include dyeing textile fibres, colouring different materials, plastics, biological medical studies, lasers, liquid crystalline displays, electro-optical devices and ink-jet printers in high-technology areas (Gregory, 1991 ▸). The conversion from the *trans* to the *cis* form in azo compounds can lead to photochromism. Photochromic compounds are of great inter­est for the control and measurement of radiation intensity, optical computers and display systems (Dürr & Bouas-Laurent, 1990 ▸), and for potential applications in mol­ecular electronic devices (Martin *et al.*, 1995 ▸). Schiff bases often exhibit various biological activities, including anti­bacterial, anti­cancer, anti-inflammatory and anti­toxic properties (Lozier *et al.*, 1975 ▸). The present work is part of an ongoing structural study of heterocyclic compounds (Faizi *et al.*, 2016 ▸, 2017 ▸) and excited state proton-transfer compounds and fluorescent chemosensors (Faizi *et al.*, 2018 ▸; Kumar *et al.*, 2018 ▸; Mukherjee *et al.*, 2018 ▸). In the present work, we report the synthesis, crystal structure and Hirshfeld surface analysis of the title compound, (*E*)-2-[1-hy­droxy-2-(pyridin-2-yl)eth­yl]-4-[2-(4-meth­oxy­phen­yl)diazen-1-yl]phenol.
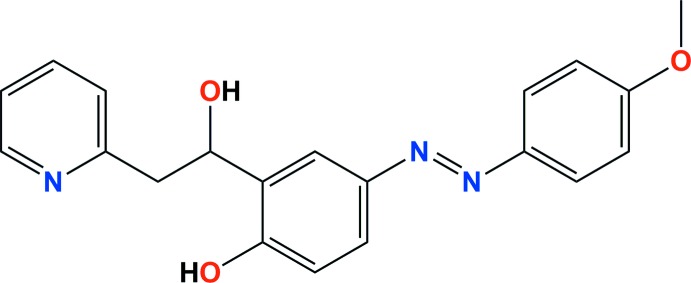


## Structural commentary   

**Figure 1:**
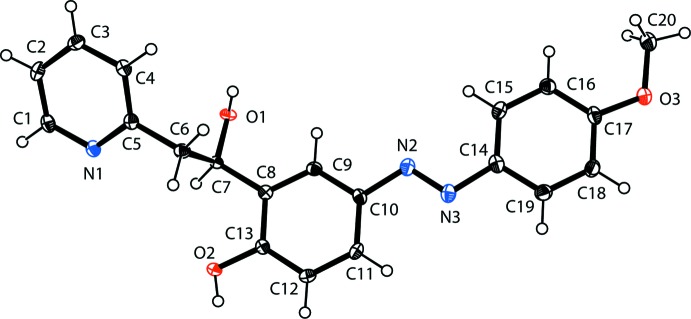
The mol­ecular structure of the title compound, with the atom labelling. Displacement ellipsoids are drawn at the 40% probability level.

The mol­ecular structure of the title compound is illustrated in Fig. 1 ▸. The configuration about the azo N=N bond is *E*, and the N2=N3 bond length is 1.256 (2) Å. The mol­ecule is non-planar, with the central benzene ring (C8–C13) being inclined to the pyridine ring (N1/C1–C5) by 31.43 (8)° and to the outer 4-meth­oxy­phenyl ring (C14–C19) by 4.73 (8)°.

## Supra­molecular features   

**Figure 2:**
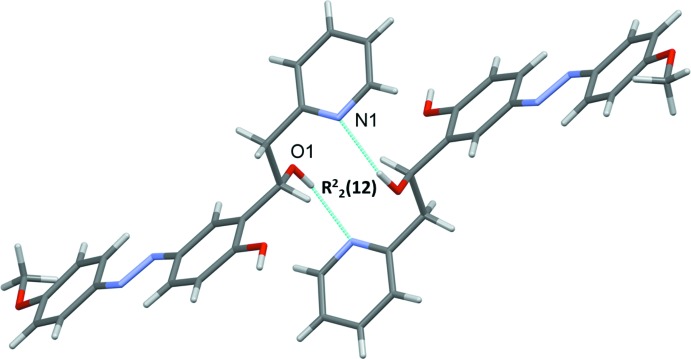
A view of the inversion dimer forming an 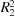
(12) ring motif; see Table 1 ▸ for details of the hydrogen-bonding (dashed lines) inter­actions involved.

**Figure 3:**
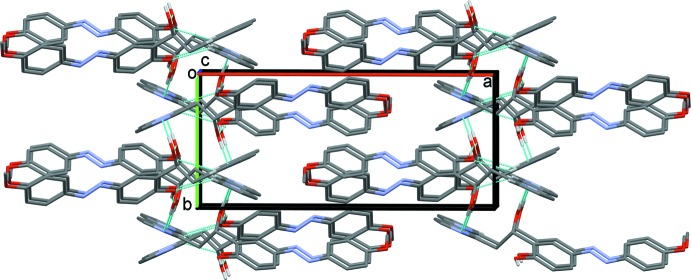
A view along the *c* axis of the crystal packing of the title compound. For clarity, H atoms not involved in hydrogen bonding (dashed lines, see Table 1 ▸) have been omitted.

**Figure 4:**
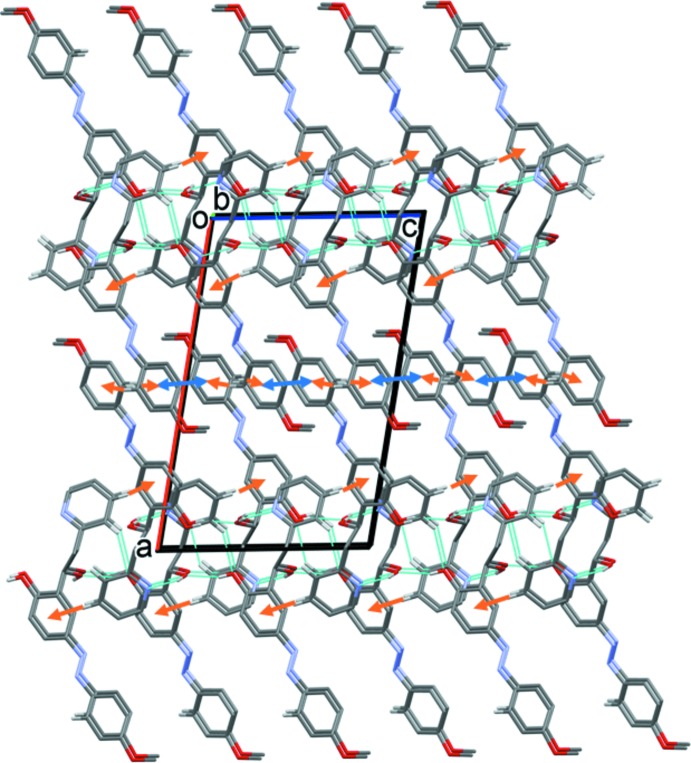
A view along the *b* axis of the crystal packing of the title compound. For clarity, H atoms not involved in hydrogen bonding (dashed lines, see Table 1 ▸) have been omitted. The C—H⋯π inter­actions are represented by brown arrows and the offset π–π inter­actions by blue double arrows.

In the crystal, mol­ecules are linked by pairs of O—H⋯N hydrogen bonds, forming inversion dimers with an 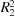
(12) ring motif (Table 1 ▸ and Fig. 2 ▸). The dimers are linked by O—H⋯O and C—H⋯O hydrogen bonds, forming undulating layers lying parallel to the *ac* plane (Fig. 3 ▸ and Table 1 ▸). There are C—H⋯π inter­actions present within the layers and between the layers, leading to the formation of a supra­molecular framework (Table 1 ▸ and Fig. 4 ▸). The layers are also linked by offset π–π inter­actions, involving inversion-related 4-meth­oxy­phenol rings, which strengthen the supra­molecular framework [*Cg*3⋯*Cg*3^vi^ = 3.584 (2) Å, inter­planar distance = 3.416 (2) Å, offset = 1.085 Å; *Cg*3 is the centroid of the C14–C19 ring; symmetry code: (vi) −*x* + 1, −*y* + 1, −*z* + 1].

## Database survey   

A search of the Cambridge Structural Database (CSD, V5.40, update of February 2019; Groom *et al.*, 2016 ▸) for compounds containing the 4-[(4-meth­oxy­phen­yl)diazen­yl]phenol skeleton gave 14 hits. There are five compounds that closely resemble the title compound, namely (*E*)-2-acetyl-4-(4-meth­oxy­phenyl­diazen­yl)phenol (CSD refcode AQIDIO; Yazici *et al.*, 2011 ▸), 2-hy­droxy-5-[(*E*)-(4-meth­oxy­phen­yl)diazen­yl]benzoic acid (FUGYIP; Basu Baul *et al.*, 2000 ▸), 4-[(*E*)-(4-meth­oxy­phen­yl)diazen­yl]-2-((*E*)-{[4-(phenyl­amino)­phen­yl]imino} meth­yl)phenol (MANTON; Faizi *et al.*, 2017 ▸), 2,6-dimethyl-4-(4-meth­oxy­phenyl­diazen­yl)phenol (PAHFUA; Kocaokutgen *et al.*, 2004 ▸) and 2-methyl-4-(4-meth­oxy­phenyl­azo)phenol (VEVKEN; İskeleli *et al.*, 2006 ▸). In all five compounds, the configuration about the N=N bond is *E*, and the dihedral angles between the 4-meth­oxy­phenyl ring and the other aryl ring are *ca* 3.04, 5.43, 11.61, 8.34 and 16.01°, respectively. In the title compound, this dihedral angle is 4.73 (8)°, similar to that in AQIDIO and FUGYIP.

## Hirshfeld surface analysis and two-dimensional fingerprint plots   

The Hirshfeld surface analysis (Spackman & Jayatilaka, 2009 ▸) and the associated two-dimensional fingerprint plots (McKinnon *et al.*, 2007 ▸) were performed with *CrystalExplorer17* (Turner *et al.*, 2017 ▸). The reader is referred to a recent article by Tiekink and collaborators (Tan *et al.*, 2019 ▸) who have published an excellent explanation of the use of Hirshfeld surface analysis and other calculations to study mol­ecular packing.

**Figure 5:**
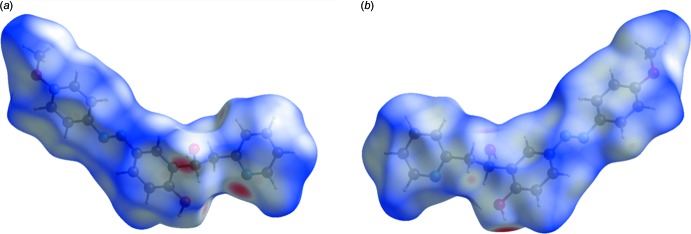
Two views, (*a*) front and (*b*) back, of the Hirshfeld surface of the title compound mapped over *d*_norm_.

**Figure 6:**
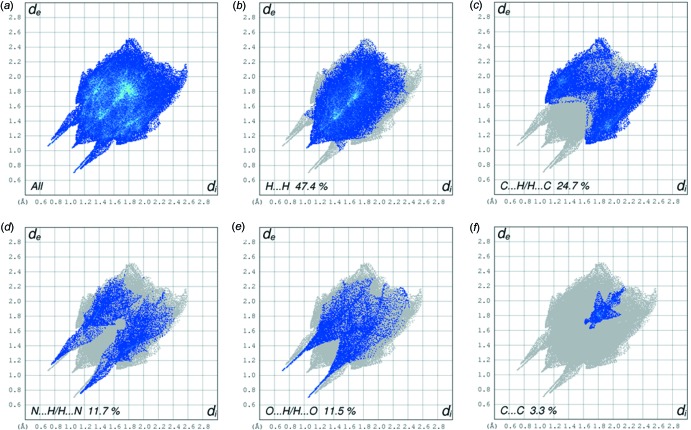
(*a*) The full two-dimensional fingerprint plot for the title compound, and the two-dimensional fingerprint plots delineated into (*b*) H⋯H, (*c*) C⋯H/H⋯C, (*d*) N⋯H/H⋯N, (*e*) O⋯H/H⋯O, (*f*) C⋯C contacts.

Two views, front and back, of the Hirshfeld surface of the title compound mapped over *d*_norm_ are given in Fig. 5 ▸, and the two-dimensional fingerprint plots are given in Fig. 6 ▸. The latter reveals that the principal inter­molecular contacts are, as is often the case, H⋯H at 47.4% (Fig. 6 ▸ *b*). This is followed by the H⋯C/C⋯H contacts at 24.7% (Fig. 6 ▸ *c*), related to the C—H⋯π inter­actions (see Table 1 ▸ for details). The classical O—H⋯N hydrogen bonds (Table 1 ▸) contribute, *via* N⋯H/H⋯N contacts (11.7%; Fig. 6 ▸ *d*), while the classical O—H⋯O and non-classical C—H⋯O hydrogen bonds (Table 1 ▸) contribute, *via* O⋯H/H⋯O contacts (11.5%; Fig. 6 ▸ *e*). The C⋯C contacts contribute only 3.3% (Fig. 6 ▸ *f*), but are significant when analysing the offset π–π inter­actions in the crystal (see §3. *Supra­molecular features*) and the formation of the supra­molecular framework.

## Synthesis and crystallization   

The title compound was prepared by adding *n*-butyl­lithium (4.91 ml, 12.29 mmol, 2.5 *M* in cyclo­hexa­ne) to a solution of 2-picoline (1 ml, 10.24 mmol) in anhydrous THF (25 ml) cooled at 195 K. The orange mixture was left to warm up to 143 K and then 5-(4-meth­oxy­phenyl­azo)salicyaldehyde (MPS) (2.00 g, 8.53 mmol) dissolved in THF (10 ml) was added, giving a yellow solution. The solution was then stirred for 2 h at room temperature. The reaction was quenched by the addition of an aqueous saturated solution of ammonium chloride (50 ml), and the product was extracted with diethyl ether. It was then dried over MgSO_4_ and purified by column chromatography (cyclo­hexa­ne/ethyl acetate 9/1) to give a yellow solid (1.10 g, 3.36 mmol, yield: 60%). Yellow needle-like crystals of the title compound were obtained by slow evaporation of a solution in methanol.

Spectroscopic and analytical data: Yellow solid: *R*_f_ = 0.43 (cyclo­hexa­ne/ethyl acetate = 9/1); IR ν_max_ (KBr, cm^−1^): 3170, 2837, 1596, 1500, 1480, 1440, 1428, 1339, 1281, 1257, 1206, 1178, 1140, 1103, 1052, 1032, 1005, 905, 869, 841, 824, 773, 730, 652, 570, 531, 493; ^1^H NMR (500 MHz, CDCl_3_) δ 3.14 (*dd*, 1H, *J* = 2.1, 15.8Hz), 3.44–3.49 (*m*, 1H), 3.88 (*s*, 3H), 5.46–5.49 (*m*, 1H), 6.98–7.01 (*m*, 3H), 7.21 (*d*, 1H, *J* = 7.6 Hz), 7.62–7.63 (*m*, 1H), 7.69–7.73 (*m*, 1H), 7.78 (*dd*, 1H, *J* =2.5, 8.6 Hz), 7.84–7.86 (*m*, 2H); ^13^C NMR (125 MHz, CDCl_3_) δ 42.7, 55.6,75.1, 114.2, 118.1, 121.4, 122.4, 124.1, 124.2, 124.3, 126.6, 137.7, 146.2, 147.1, 148.0, 159.2, 159.6, 161.5; HRMS (ESI) for C_20_H_20_N_3_O_3_ (*M* + H^+^): calculated 350.1504, found: 350.1507.

## Refinement   

Crystal data, data collection and structure refinement details are summarized in Table 2 ▸. The OH and C-bound H atoms were included in calculated positions and treated as riding atoms: O—H = 0.82 Å and C—H = 0.93–0.98 Å, with *U*_iso_(H) = 1.5*U*_eq_(O-hydroxyl and C-meth­yl) and 1.2*U*_eq_(C) for other H atoms.

## Supplementary Material

Crystal structure: contains datablock(s) I, Global. DOI: 10.1107/S2056989019004377/su5489sup1.cif

Structure factors: contains datablock(s) I. DOI: 10.1107/S2056989019004377/su5489Isup2.hkl

Supporting information file. DOI: 10.1107/S2056989019004377/su5489Isup3.cml

CCDC reference: 959013

Additional supporting information:  crystallographic information; 3D view; checkCIF report

## Figures and Tables

**Table 1:** Hydrogen-bond geometry (Å, °) *Cg*2 and *Cg*3 are the centroids of rings C8–C13 and C14–C19, respectively.

*D*—H⋯*A*	*D*—H	H⋯*A*	*D*⋯*A*	*D*—H⋯*A*
O1—H1⋯N1^i^	0.82	2.04	2.801 (2)	154
O2—H2⋯O1^ii^	0.82	1.91	2.686 (2)	158
C4—H4⋯O2^iii^	0.93	2.47	3.165 (2)	132
C3—H3⋯*Cg*2^iv^	0.93	2.82	3.593 (3)	141
C19—H19⋯*Cg*3^v^	0.93	2.98	3.841 (3)	155

Symmetry codes: (i) 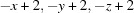; (ii) 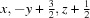; (iii) 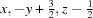; (iv) 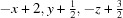; (v) 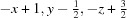.

**Table 2:** Experimental details

Crystal data	
Chemical formula	C_20_H_19_N_3_O_3_
*M*_r_	349.38
Crystal system, space group	Monoclinic, *P*2_1_/*c*
Temperature (K)	296
*a*, *b*, *c* (Å)	18.451 (5), 8.169 (5), 11.591 (5)
β (°)	100.059 (5)
*V* (Å^3^)	1720.2 (14)
*Z*	4
Radiation type	Mo *K*α
μ (mm^−1^)	0.09
Crystal size (mm)	0.30 × 0.25 × 0.20

Data collection	
Diffractometer	Bruker APEXII CCD area detector
Absorption correction	Multi-scan (*SADABS*; Bruker, 2003 ▸)
*T*_min_, *T*_max_	0.281, 0.397
No. of measured, independent and observed [*I* > 2σ(*I*)] reflections	12516, 3381, 2169
*R*_int_	0.056
(sin θ/λ)_max_ (Å^−1^)	0.617

Refinement	
*R*[*F* ^2^ > 2σ(*F* ^2^)], *wR*(*F* ^2^), *S*	0.045, 0.100, 1.02
No. of reflections	3381
No. of parameters	238
H-atom treatment	H-atom parameters constrained
Δρ_max_, Δρ_min_ (e Å^−3^)	0.23, −0.19

Computer programs: *APEX2* and *SAINT* (Bruker, 2003 ▸), *SHELXS2018* (Sheldrick, 2008 ▸), *PLATON* (Spek, 2009 ▸), *SHELXL2018* (Sheldrick, 2015 ▸), *Mercury* (Macrae *et al.*, 2008 ▸), *PLATON* (Spek, 2009 ▸) and *publCIF* (Westrip, 2010 ▸).

## supplementary crystallographic information

### Crystal data

**Table:**

C_20_H_19_N_3_O_3_	*F*(000) = 736
*M**_r_* = 349.38	*D*_x_ = 1.349 Mg m^−^^3^
Monoclinic, *P*2_1_/*c*	Mo *K*α radiation, λ = 0.71073 Å
*a* = 18.451 (5) Å	Cell parameters from 1490 reflections
*b* = 8.169 (5) Å	θ = 3.7–26.0°
*c* = 11.591 (5) Å	µ = 0.09 mm^−^^1^
β = 100.059 (5)°	*T* = 296 K
*V* = 1720.2 (14) Å^3^	Needle, yellow
*Z* = 4	0.30 × 0.25 × 0.20 mm

### Data collection

**Table:**

Bruker APEXII CCD area detector diffractometer	3381 independent reflections
Radiation source: sealed tube	2169 reflections with *I* > 2σ(*I*)
Graphite monochromator	*R*_int_ = 0.056
phi and ω scans	θ_max_ = 26.0°, θ_min_ = 2.2°
Absorption correction: multi-scan (SADABS; Bruker, 2003)	*h* = −22→13
*T*_min_ = 0.281, *T*_max_ = 0.397	*k* = −10→10
12516 measured reflections	*l* = −14→14

### Refinement

**Table:**

Refinement on *F*^2^	Primary atom site location: structure-invariant direct methods
Least-squares matrix: full	Secondary atom site location: difference Fourier map
*R*[*F*^2^ > 2σ(*F*^2^)] = 0.045	Hydrogen site location: inferred from neighbouring sites
*wR*(*F*^2^) = 0.100	H-atom parameters constrained
*S* = 1.02	*w* = 1/[σ^2^(*F*_o_^2^) + (0.0428*P*)^2^ + 0.2309*P*] where *P* = (*F*_o_^2^ + 2*F*_c_^2^)/3
3381 reflections	(Δ/σ)_max_ = 0.001
238 parameters	Δρ_max_ = 0.23 e Å^−^^3^
0 restraints	Δρ_min_ = −0.19 e Å^−^^3^

### Special details

**Table:**

Geometry. All esds (except the esd in the dihedral angle between two l.s. planes) are estimated using the full covariance matrix. The cell esds are taken into account individually in the estimation of esds in distances, angles and torsion angles; correlations between esds in cell parameters are only used when they are defined by crystal symmetry. An approximate (isotropic) treatment of cell esds is used for estimating esds involving l.s. planes.

### Fractional atomic coordinates and isotropic or equivalent isotropic displacement parameters (Å^2^)

**Table:**

	*x*	*y*	*z*	*U*_iso_*/*U*_eq_	
O1	0.92494 (7)	0.95556 (14)	0.84820 (10)	0.0173 (3)	
H1	0.911689	1.038088	0.879019	0.026*	
O2	0.92659 (7)	0.63827 (16)	1.12657 (10)	0.0192 (3)	
H2	0.917704	0.591870	1.185268	0.029*	
O3	0.36888 (7)	0.68602 (17)	0.45972 (11)	0.0273 (4)	
N1	1.11161 (8)	0.82930 (19)	0.98278 (13)	0.0185 (4)	
N2	0.68013 (9)	0.68483 (19)	0.77772 (13)	0.0211 (4)	
N3	0.62436 (9)	0.6015 (2)	0.78765 (13)	0.0215 (4)	
C5	1.06621 (10)	0.7859 (2)	0.88320 (16)	0.0165 (4)	
C4	1.08607 (10)	0.8094 (2)	0.77434 (16)	0.0180 (4)	
H4	1.053929	0.778909	0.706776	0.022*	
C1	1.17713 (11)	0.8933 (2)	0.97259 (17)	0.0208 (5)	
H1A	1.209217	0.920515	1.041003	0.025*	
C2	1.20001 (11)	0.9212 (2)	0.86736 (17)	0.0220 (5)	
H2A	1.245666	0.967932	0.864834	0.026*	
C3	1.15317 (10)	0.8778 (2)	0.76592 (16)	0.0205 (5)	
H3	1.166638	0.894338	0.693146	0.025*	
C6	0.99387 (10)	0.7089 (2)	0.89526 (16)	0.0184 (4)	
H6A	1.003179	0.620943	0.952107	0.022*	
H6B	0.972356	0.660762	0.820624	0.022*	
C7	0.93790 (10)	0.8263 (2)	0.93306 (15)	0.0155 (4)	
H7	0.958956	0.873197	1.009369	0.019*	
C8	0.86746 (10)	0.7372 (2)	0.94404 (15)	0.0150 (4)	
C9	0.80465 (10)	0.7454 (2)	0.85966 (16)	0.0171 (4)	
H9	0.805051	0.809928	0.793644	0.021*	
C10	0.74100 (10)	0.6607 (2)	0.87024 (15)	0.0167 (4)	
C11	0.74037 (10)	0.5601 (2)	0.96750 (16)	0.0200 (5)	
H11	0.698601	0.499720	0.974301	0.024*	
C12	0.80215 (10)	0.5512 (2)	1.05329 (15)	0.0176 (4)	
H12	0.801859	0.485124	1.118536	0.021*	
C13	0.86500 (10)	0.6403 (2)	1.04309 (15)	0.0155 (4)	
C14	0.56281 (10)	0.6283 (2)	0.69611 (16)	0.0199 (5)	
C15	0.56281 (10)	0.7290 (2)	0.59953 (16)	0.0208 (5)	
H15	0.605862	0.782243	0.589646	0.025*	
C16	0.49943 (11)	0.7506 (2)	0.51820 (17)	0.0216 (5)	
H16	0.499724	0.818338	0.453772	0.026*	
C17	0.43502 (10)	0.6707 (2)	0.53292 (16)	0.0213 (5)	
C18	0.43565 (11)	0.5659 (2)	0.62726 (16)	0.0235 (5)	
H18	0.393131	0.509452	0.635834	0.028*	
C19	0.49901 (10)	0.5451 (2)	0.70808 (16)	0.0228 (5)	
H19	0.499085	0.474861	0.771220	0.027*	
C20	0.36725 (11)	0.7824 (3)	0.35657 (17)	0.0298 (5)	
H20A	0.318289	0.782488	0.311822	0.045*	
H20B	0.400634	0.736834	0.310239	0.045*	
H20C	0.381746	0.892552	0.378279	0.045*	

### Atomic displacement parameters (Å^2^)

**Table:**

	*U*^11^	*U*^22^	*U*^33^	*U*^12^	*U*^13^	*U*^23^
O1	0.0236 (8)	0.0129 (7)	0.0162 (7)	0.0019 (6)	0.0053 (6)	0.0002 (6)
O2	0.0192 (7)	0.0238 (8)	0.0139 (7)	−0.0022 (6)	0.0009 (6)	0.0042 (6)
O3	0.0176 (8)	0.0376 (9)	0.0252 (8)	−0.0018 (7)	−0.0007 (6)	0.0062 (7)
N1	0.0175 (9)	0.0192 (9)	0.0187 (9)	0.0023 (7)	0.0028 (7)	−0.0024 (7)
N2	0.0180 (9)	0.0235 (9)	0.0221 (9)	−0.0016 (8)	0.0044 (7)	−0.0040 (8)
N3	0.0183 (9)	0.0222 (9)	0.0239 (9)	−0.0016 (8)	0.0029 (8)	−0.0038 (7)
C5	0.0169 (11)	0.0125 (9)	0.0197 (10)	0.0038 (8)	0.0019 (9)	−0.0002 (8)
C4	0.0185 (11)	0.0169 (10)	0.0180 (10)	0.0020 (9)	0.0011 (8)	−0.0029 (8)
C1	0.0178 (11)	0.0213 (11)	0.0214 (11)	0.0014 (9)	−0.0020 (9)	−0.0053 (9)
C2	0.0155 (11)	0.0189 (10)	0.0326 (12)	−0.0017 (9)	0.0068 (10)	−0.0011 (9)
C3	0.0215 (11)	0.0195 (10)	0.0222 (11)	0.0026 (9)	0.0082 (9)	0.0030 (9)
C6	0.0207 (11)	0.0163 (10)	0.0185 (10)	0.0018 (9)	0.0043 (9)	0.0020 (8)
C7	0.0168 (10)	0.0168 (10)	0.0125 (9)	0.0002 (8)	0.0014 (8)	0.0024 (8)
C8	0.0167 (11)	0.0124 (10)	0.0158 (10)	0.0005 (8)	0.0028 (9)	−0.0029 (8)
C9	0.0189 (11)	0.0182 (10)	0.0150 (10)	0.0018 (9)	0.0052 (8)	−0.0007 (8)
C10	0.0160 (11)	0.0180 (10)	0.0153 (10)	0.0032 (9)	0.0007 (8)	−0.0034 (8)
C11	0.0160 (11)	0.0209 (11)	0.0241 (11)	−0.0042 (9)	0.0063 (9)	−0.0019 (9)
C12	0.0217 (11)	0.0175 (10)	0.0150 (10)	−0.0001 (9)	0.0067 (9)	0.0010 (8)
C13	0.0160 (11)	0.0150 (10)	0.0156 (10)	0.0036 (9)	0.0029 (9)	−0.0027 (8)
C14	0.0187 (11)	0.0205 (10)	0.0201 (11)	0.0035 (9)	0.0019 (9)	−0.0054 (9)
C15	0.0166 (11)	0.0206 (10)	0.0263 (11)	−0.0020 (9)	0.0066 (9)	−0.0045 (9)
C16	0.0212 (11)	0.0245 (12)	0.0191 (11)	0.0009 (9)	0.0032 (9)	−0.0011 (9)
C17	0.0168 (11)	0.0268 (12)	0.0192 (11)	0.0002 (9)	0.0001 (9)	−0.0068 (9)
C18	0.0185 (11)	0.0281 (11)	0.0245 (11)	−0.0049 (9)	0.0052 (9)	−0.0018 (9)
C19	0.0229 (12)	0.0246 (11)	0.0215 (11)	−0.0004 (10)	0.0059 (9)	0.0015 (9)
C20	0.0236 (12)	0.0348 (13)	0.0286 (12)	−0.0001 (10)	−0.0021 (10)	0.0049 (10)

### Geometric parameters (Å, º)

**Table:**

O1—C7	1.435 (2)	C7—H7	0.9800
O1—H1	0.8200	C8—C9	1.381 (2)
O2—C13	1.358 (2)	C8—C13	1.401 (2)
O2—H2	0.8200	C9—C10	1.387 (3)
O3—C17	1.365 (2)	C9—H9	0.9300
O3—C20	1.427 (2)	C10—C11	1.396 (3)
N1—C1	1.341 (2)	C11—C12	1.378 (2)
N1—C5	1.350 (2)	C11—H11	0.9300
N2—N3	1.256 (2)	C12—C13	1.392 (3)
N2—C10	1.425 (2)	C12—H12	0.9300
N3—C14	1.429 (2)	C14—C19	1.387 (3)
C5—C4	1.387 (3)	C14—C15	1.389 (3)
C5—C6	1.504 (3)	C15—C16	1.379 (3)
C4—C3	1.377 (3)	C15—H15	0.9300
C4—H4	0.9300	C16—C17	1.392 (3)
C1—C2	1.378 (3)	C16—H16	0.9300
C1—H1A	0.9300	C17—C18	1.388 (3)
C2—C3	1.378 (3)	C18—C19	1.375 (3)
C2—H2A	0.9300	C18—H18	0.9300
C3—H3	0.9300	C19—H19	0.9300
C6—C7	1.528 (3)	C20—H20A	0.9600
C6—H6A	0.9700	C20—H20B	0.9600
C6—H6B	0.9700	C20—H20C	0.9600
C7—C8	1.514 (2)		
			
C7—O1—H1	109.5	C10—C9—H9	119.0
C13—O2—H2	109.5	C9—C10—C11	119.44 (17)
C17—O3—C20	117.19 (15)	C9—C10—N2	115.63 (17)
C1—N1—C5	117.47 (16)	C11—C10—N2	124.93 (17)
N3—N2—C10	114.01 (16)	C12—C11—C10	119.43 (17)
N2—N3—C14	113.99 (16)	C12—C11—H11	120.3
N1—C5—C4	121.23 (17)	C10—C11—H11	120.3
N1—C5—C6	117.29 (16)	C11—C12—C13	120.52 (17)
C4—C5—C6	121.47 (17)	C11—C12—H12	119.7
C3—C4—C5	120.27 (18)	C13—C12—H12	119.7
C3—C4—H4	119.9	O2—C13—C12	122.62 (16)
C5—C4—H4	119.9	O2—C13—C8	116.61 (16)
N1—C1—C2	124.26 (18)	C12—C13—C8	120.77 (16)
N1—C1—H1A	117.9	C19—C14—C15	119.35 (17)
C2—C1—H1A	117.9	C19—C14—N3	115.39 (17)
C1—C2—C3	117.98 (18)	C15—C14—N3	125.25 (18)
C1—C2—H2A	121.0	C16—C15—C14	120.48 (18)
C3—C2—H2A	121.0	C16—C15—H15	119.8
C4—C3—C2	118.78 (18)	C14—C15—H15	119.8
C4—C3—H3	120.6	C15—C16—C17	119.75 (18)
C2—C3—H3	120.6	C15—C16—H16	120.1
C5—C6—C7	114.81 (16)	C17—C16—H16	120.1
C5—C6—H6A	108.6	O3—C17—C18	115.46 (18)
C7—C6—H6A	108.6	O3—C17—C16	124.79 (18)
C5—C6—H6B	108.6	C18—C17—C16	119.75 (18)
C7—C6—H6B	108.6	C19—C18—C17	120.14 (19)
H6A—C6—H6B	107.5	C19—C18—H18	119.9
O1—C7—C8	111.64 (15)	C17—C18—H18	119.9
O1—C7—C6	107.77 (14)	C18—C19—C14	120.47 (18)
C8—C7—C6	110.83 (15)	C18—C19—H19	119.8
O1—C7—H7	108.8	C14—C19—H19	119.8
C8—C7—H7	108.8	O3—C20—H20A	109.5
C6—C7—H7	108.8	O3—C20—H20B	109.5
C9—C8—C13	117.68 (17)	H20A—C20—H20B	109.5
C9—C8—C7	122.90 (16)	O3—C20—H20C	109.5
C13—C8—C7	119.42 (16)	H20A—C20—H20C	109.5
C8—C9—C10	122.10 (17)	H20B—C20—H20C	109.5
C8—C9—H9	118.9		
			
C10—N2—N3—C14	178.77 (15)	C9—C10—C11—C12	2.2 (3)
C1—N1—C5—C4	−1.1 (3)	N2—C10—C11—C12	−177.81 (17)
C1—N1—C5—C6	177.95 (16)	C10—C11—C12—C13	−0.5 (3)
N1—C5—C4—C3	0.1 (3)	C11—C12—C13—O2	178.50 (16)
C6—C5—C4—C3	−178.93 (17)	C11—C12—C13—C8	−1.8 (3)
C5—N1—C1—C2	1.8 (3)	C9—C8—C13—O2	−177.94 (15)
N1—C1—C2—C3	−1.3 (3)	C7—C8—C13—O2	2.6 (2)
C5—C4—C3—C2	0.3 (3)	C9—C8—C13—C12	2.3 (3)
C1—C2—C3—C4	0.2 (3)	C7—C8—C13—C12	−177.13 (16)
N1—C5—C6—C7	71.7 (2)	N2—N3—C14—C19	−176.48 (16)
C4—C5—C6—C7	−109.2 (2)	N2—N3—C14—C15	3.4 (3)
C5—C6—C7—O1	58.2 (2)	C19—C14—C15—C16	2.2 (3)
C5—C6—C7—C8	−179.36 (15)	N3—C14—C15—C16	−177.72 (18)
O1—C7—C8—C9	18.4 (2)	C14—C15—C16—C17	−0.1 (3)
C6—C7—C8—C9	−101.8 (2)	C20—O3—C17—C18	−175.10 (17)
O1—C7—C8—C13	−162.15 (15)	C20—O3—C17—C16	4.5 (3)
C6—C7—C8—C13	77.7 (2)	C15—C16—C17—O3	178.39 (17)
C13—C8—C9—C10	−0.6 (3)	C15—C16—C17—C18	−2.1 (3)
C7—C8—C9—C10	178.84 (17)	O3—C17—C18—C19	−178.22 (17)
C8—C9—C10—C11	−1.7 (3)	C16—C17—C18—C19	2.2 (3)
C8—C9—C10—N2	178.37 (16)	C17—C18—C19—C14	−0.1 (3)
N3—N2—C10—C9	177.37 (16)	C15—C14—C19—C18	−2.0 (3)
N3—N2—C10—C11	−2.6 (3)	N3—C14—C19—C18	177.85 (17)

### Hydrogen-bond geometry (Å, º)

*Cg*2 and *Cg*3 are the centroids of rings C8–C13 and C14–C19, respectively.

**Table:**

*D*—H···*A*	*D*—H	H···*A*	*D*···*A*	*D*—H···*A*
O1—H1···N1^i^	0.82	2.04	2.801 (2)	154
O2—H2···O1^ii^	0.82	1.91	2.686 (2)	158
C4—H4···O2^iii^	0.93	2.47	3.165 (2)	132
C3—H3···*Cg*2^iv^	0.93	2.82	3.593 (3)	141
C19—H19···*Cg*3^v^	0.93	2.98	3.841 (3)	155

Symmetry codes: (i) −*x*+2, −*y*+2, −*z*+2; (ii) *x*, −*y*+3/2, *z*+1/2; (iii) *x*, −*y*+3/2, *z*−1/2; (iv) −*x*+2, *y*+1/2, −*z*+3/2; (v) −*x*+1, *y*−1/2, −*z*+3/2.
